# Temporal changes in magnetic resonance imaging in the *mdx* mouse

**DOI:** 10.1186/1756-0500-6-262

**Published:** 2013-07-09

**Authors:** Stephen JP Pratt, Su Xu, Roger J Mullins, Richard M Lovering

**Affiliations:** 1 Department of Orthopaedics, University of Maryland, School of Medicine AHB, Rm 540, 100 Penn St, Baltimore, MD 21201, USA; 2 Diagnostic Radiology and Nuclear Medicine, University of Maryland, School of Medicine, 22 S. Greene St, Baltimore, MD 21201, USA

**Keywords:** *In vivo* magnetic resonance imaging, *mdx*

## Abstract

**Background:**

Duchenne muscular dystrophy (DMD) is characterized clinically by severe, progressive loss of skeletal muscle. The phenotype is much less severe in the *mdx* mouse model of DMD than that seen in patients with DMD. However, a “critical period” has been described for the *mdx* mouse, during which there is a peak in muscle weakness and degeneration/regeneration between the 2^nd^ and 5^th^ weeks of life. A number of studies have employed small animal magnetic resonance imaging (MRI) to examine skeletal muscle in various dystrophic models, but such studies represent a snapshot in time rather than a longitudinal view.

**Results:**

The *in vivo* cross-sectional T_2_-weighted image of the healthy (wild type, WT) muscles is homogeneously dark and this homogeneity does not change with time, as there is no disease. We, and others, have shown marked changes in MRI in dystrophic muscle, with multiple, unevenly distributed focal hyperintensities throughout the bulk of the muscles. Here we monitored an *mdx* mouse using MRI from 5 to 80 weeks of age. Temporal MRI scans show an increase in heterogeneity shortly after the critical period, at 9 and 13 weeks of age, with a decrease in heterogeneity thereafter. The 4.3-fold increase in percent heterogeneity at week 9 and 13 is consistent with the notion of an early critical period described for *mdx* mice.

**Conclusions:**

Age is a significant variable in quantitative MR studies of the *mdx* mouse. The *mdx* mouse is typically studied during the critical period, at a time that most closely mimics the DMD pathology, but the preliminary findings here, albeit based on imaging only one *mdx* mouse over time, suggest that the changes in MRI can occur shortly after this period, when the muscles are still recovering.

## Background

Plain films, or x-rays, have limited use for imaging muscle pathology, unless heterotopic bone formation has occurred within the muscle. Unlike x-rays, magnetic resonance imaging (MRI) offers superb tissue contrast and has high sensitivity to the hemorrhage and edema that accompany muscle damage. This, together with its non-invasive nature, makes MRI a potentially ideal technique for evaluating muscular dystrophies. As non-invasive technology continues to improve and imaging such as MRI becomes more commonplace, it is likely to play a greater role for diagnosis, prognosis, and in rehabilitation planning [1].

Duchenne muscular dystrophy (DMD) is characterized clinically by severe, progressive loss of skeletal muscle. The disease is caused by the lack of dystrophin, a large membrane-associated protein expressed in skeletal muscle and localized to the inner face of the sarcolemma [2]. Much of what is known about dystrophin structure-function is derived from studies of dystrophin-deficient animals, with the most common model being the *mdx* mouse. The DMD and the *mdx* conditions are similar in that dystrophin is missing from all muscle tissues. However, the absence of dystrophin is not equally damaging to patients with DMD and *mdx* mice; the *mdx* phenotype is much less severe than that seen with DMD. A “critical period” has been described for the *mdx* mouse, during which there is a peak in muscle weakness, myofiber necrosis and regeneration between the 2^nd^ and 5^th^ weeks of life [3-7]. Throughout the rest of their lifespan, *mdx* mice show marked susceptibility to contraction-induced injury, but only minimal weakness. Mechanical function is much less compromised than in DMD, so much so that the lifespan of the *mdx* mouse can be normal.

A number of studies have employed small animal MRI to examine skeletal muscle in various dystrophic models [8-13]. Evaluation of T_2_ has been reported in both human and murine forms of muscular dystrophy, but such studies represent snapshots in time rather than a longitudinal view. We were curious to know if the well-established MRI changes seen during the critical period continued throughout the lifespan of *mdx* mice. We examined MRI images of dystrophic hindlimb muscles from an *mdx* mouse over time. Even though MRI has been used to study the *mdx* mouse [10,11,14,15], this is, to the best of our knowledge, the first time a longitudinal view has been examined. The goal was to non-invasively monitor the progression of muscular dystrophy in the *mdx* animal model.

## Materials and methods

### Animals

The protocol was approved by the University of Maryland Institutional Animal Care & Use Committee. One wild type mouse (WT, C57BL/10ScSn) and one dystrophic mouse (*mdx*, C57BLScSn-DMD^*mdx*^) were acquired (Jackson Laboratory, Bar Harbor, ME) and used for imaging.

### In vivo MRI experiments

MRI was performed over the course of 80 weeks, starting at 5 weeks of age and in increments of at least every 4 weeks. Gaps in scanning greater than 4 weeks are indicated by breaks in figure axes. MRI studies were performed on a Bruker Biospec 7.0 Tesla 30-cm horizontal bore scanner using Paravision 5.1 software (Bruker Biospin MRI GmbH, Germany). A Bruker four-element ^1^H surface coil array was used as the receiver and a Bruker 72 mm linear-volume coil as the transmitter. The mouse was anesthetized in an animal induction chamber with a gas mixture of O_2_ (1 L/min) and isoflurane (3%). The animal was then placed supine on a custom made body holder bed and the radio frequency coil was positioned and fixed with surgical tape in the region of interest on the animal leg. After the animal was moved into the center of the magnet, the isoflurane level was maintained 1.0 to 1.5% for the remainder of the experiment. An MR-compatible small-animal monitoring and gating system (SA Instruments, Inc., New York, NY) was used to monitor animal respiration rate and body temperature. Mouse body temperature was maintained at 36 – 37°C using a warm water circulator. Three-slice (axial, mid-sagittal, and coronal) scout rapid acquisition with fast low angle shot MR imaging (FLASH) was used to localize the leg. High resolution T_2_-weighted MRI images in the cross-sectional view between the knee and the ankle were acquired using apid acquisition with relaxation enhancement (RARE) sequence with TR/TE (repetition time/echo time) = 5000/32 ms, RARE factor = 8, field of view (FOV) = 30 × 30 mm^2^, matrix size = 250 × 250, slice thickness = 0. 5 mm without a gap, averages = 16, number of slices = 32.

### Image processing

**Figure 1:**
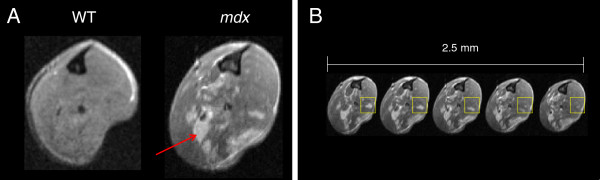
**MR imaging and measurement of heterogeneity in the mouse. A**: Representative MR images of hindlimb muscles in healthy (wild type, WT) and dystrophic (*mdx*) mice, both at 9 weeks. The signal in WT hindlimb muscles is homogeneously dark and does not change over time. Compared to WT, dystrophic muscles show heterogeneity, identified by unevenly distributed focal hyperintensities (red arrow) that contrast the dark signal characteristic of healthy muscle. **B**: The image shows consecutive 0.5 mm thick image axial slices from an *mdx* hindlimb. Such consecutive slices were used to obtain volume (mm^3^) ratios of focal hyperintensities to homogeneously dark muscle at every time point and to account for possible changes in heterogeneity along the longitudinal axis of the muscles (yellow boxes).

The high resolution cross-section T_2_-weighted images were processed using the shading correction, VOI drawing and statistics functions of Medical Image Processing, Analysis and Visualization (MIPAV v5.3.1, CIT, NIH, Bethesda, MD). Shading correction was performed using the Inhomogeneity N3 correction function, after which the interactive level-set VOI method was used to semi-automatically segment each of the high signal intensity regions inside of muscle from the rest of the muscle tissue in view. Volume was then determined using the VOI statistics generator. Percent heterogeneity was calculated using volume (mm^3^) ratios of focal hyperintensity volume to the whole muscle volume. To obtain volume and account for possible changes in heterogeneity along the long axis of the muscles (Figure 1B, yellow boxes) five consecutive MRI slices, 0.5 mm thick, were centered on the tibia and were used for imaging quantification. Figure images are of the same image slice, relative to the tibial head, for each scan time point.

## Results

**Figure 2:**
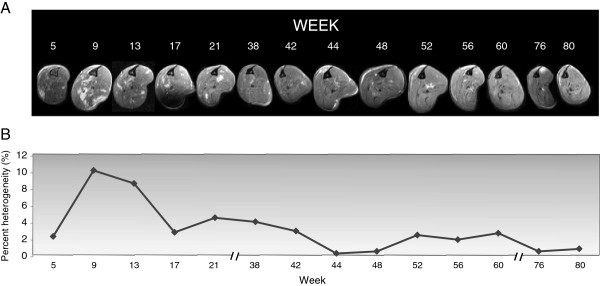
**Temporal changes in MRI heterogeneity.A**: Representative images of dystrophic (*mdx*) mouse hindlimb muscles over time. The mouse was scanned in increments of about 4 weeks, throughout the course of 20 months, starting at 5 weeks of age. Changes in the shape of the legs are a consequence of the custom-designed apparatus used to stabilize them. **B**: Line graph shows the percent of heterogeneity for each time point. Heterogeneity reaches its peak around 9 and 13 weeks of age, just after the early critical period of the *mdx* mouse. Interestingly, the drop in heterogeneity of the T_2_ signal remains relatively stable through 80 weeks of age. Timeline gaps are indicated by breaks in the axis.

The *in vivo* cross-sectional T_2_-weighted image of the healthy (WT) muscles is homogeneously dark at 7 T [16,17] (Figure 1A) and this homogeneity does not change with time, as there is no disease. By contrast, the MRI of *mdx* muscles shows heterogeneity, with multiple, unevenly distributed focal hyperintensities throughout the bulk of the muscles [11] (Figure 1A, red arrow). The data from the 14 imaging sessions (Figure 2A, from week 5 to week 80) show these unevenly distributed focal hyperintensities throughout the muscles, with a peak in this presentation occurring around 9 and 13 weeks of age (the overall shape of the leg is altered due to the custom-designed apparatus used to stabilize the legs). The *mdx* mouse is typically studied during the critical period, at a time that most closely mimics the DMD pathology. Interestingly, the heterogeneity in *mdx* muscles appears to change over time. Percent heterogeneity was quantified (Figure 2B) and shows a peak (10.3%) at 9 weeks of age with a similar peak (8.7%) at week 13. The drop in heterogeneity thereafter remains relatively stable thorough 80 weeks of age. However, there is a stepwise decline in the percent heterogeneity from weeks 5–17 (peak 10.3%), weeks 17–44 (peak 4.7%), and weeks 48–76 (peak 2.8%).

## Discussion

In 1984, animals of the C57BL/10 strain were identified that had abnormally elevated levels of serum pyruvate kinase and creatine kinase [18], suggesting the existence of a muscular dystrophy. The disease was linked to the X chromosome and the pathohistology was indicative of a primary myopathy. The mutant was named “mdx” (X-linked muscular dystrophy) and the discovery that its muscles lacked dystrophin indicated it was a homolog of DMD [19-21].

In patients with DMD, the dystrophic process is progressive from birth, while in the *mdx* mouse model, the dystrophic phenotype is most evident around the post-weaning period, when these animals initially become active. The *mdx* mouse has been used as a model for DMD for years and is still considered the most suitable mouse model for DMD [22]. While *mdx* hindlimb muscles seem to partially recover after 2–3 months, forced running (metabolic stressor) and or high-force lengthening contractions (mechanical stressor) can increase the severity of the murine phenotype to better mimic the pathology found in DMD.

In the present study, we performed *in vivo* MRI to examine differences in *mdx* hindlimb muscles of a caged *mdx* mouse over time. The data clearly show localized hyperintense regions in muscles of the *mdx* mouse, which peak near the critical period, when the muscles undergo maximal degeneration and regeneration. Interestingly, there was little change in the T_2_ signal heterogeneity from *mdx* muscles at later ages, at a time when muscle fiber splitting is greatest [23], histological integrity deteriorates [3] and muscle necrosis continues despite a slight reduction in regeneration [24]. Our findings suggest that researchers need to consider the age of *mdx* mice when designing imaging studies or evaluating MRI findings.

Muscle damage is typically assessed by T_2_-weighted MRI images, which optimize contrast between injured muscles with edema (increased signal intensity) and normal uninjured muscles (the T_1_ and T_2_ relaxation times in MRI define the manner in which protons in tissues revert back to their resting states after an initial radiofrequency pulse). In healthy muscle, injury causes edema and the resulting T_2_ signal to increase significantly. However, after some injury protocols there is a long-term persistence of this signal [25,26] well after inflammation and edema have resolved [27]. Some studies have shown that T_2_ values peak after injury, either as early as 7 hours [28] or as late as 2 days [29]. Some of these differences could be due to the mode and degree of injury, but overall such findings suggest that changes in T_2_ can reflect more than just inflammation. It has been proposed that a change in T_2_ is not necessarily due to fat or water content, but instead due to some intrinsic property of the cell, such as membrane composition or disruption [13]. Disease-related changes in contractile protein content and structure at the molecular and cellular level can influence muscle function [30] and the anabolic and catabolic stimuli that effect such changes [31] might play a yet unexamined role in contributing toward changes in the T_2_ signal (and function). McIntosh et al. [15] provided one of the first studies to use MRI to assess skeletal muscle in *mdx* mice, where the authors observed the heterogeneous signal intensity on T_2_-weighted images that is typically noted in DMD. These foci of high intensity have since been noted by others in the *mdx* hindlimb muscles [11,17]. In DMD, progressive replacement of skeletal muscle by fatty tissue might help explain the chronic heterogeneity, as fat can also appear bright in standard T_2_ MRI. However, it is now clear from MR spectroscopy studies that the heterogeneity in the T_2_ signal in *mdx* mice is not due to fatty infiltrate [11,13,32].

This short study has several limitations. While the data here are interesting, they lack measures preceding and during the critical period in the *mdx* mouse to better describe this early point in disease progression. The most obvious limitation is the use of only one dystrophic animal. Although *mdx* mice are of the same genetic background and therefore do not differ significantly from one another, this report effectively represents a longitudinal case report. If further studies corroborate our findings of increased heterogeneity limited to early time points, disease progression could vary with age, even between animals of the same strain in an identical environment. For such reasons, extrapolation-oriented conclusions regarding the population of *mdx* mice must be made with caution.

## Conclusions

*Mdx* mice have muscle pathology, but the phenotype is much less severe than that seen with DMD in humans. Because the pathological progression between the *mdx* mice and patients with DMD differs substantially, the validity of the *mdx* mouse as a model of DMD has been questioned and some have advocated for other models and/or double mutants (i.e., animals lacking dystrophin and utrophin) [33]. Our study suggests that the age of the animal is relevant when assessing skeletal muscles in the *mdx* mouse by non-invasive imaging. It also suggests the possibility that changes in T_2_ signal do not correspond to the period of time when muscle degeneration and regeneration peak. As pointed out earlier, these are preliminary results and based solely on one mouse. The histological changes and time period of the critical period for the *mdx* mouse is well described. However, a much larger sample size is required to confirm that the peak in T_2_ signal just *after* the critical period is typical of the *mdx* population, as well as to confirm the overall changes with time seen here. Continued studies into imaging of sedentary and exercised *mdx* mice will be of value in monitoring the progression of the dystrophic process and could be useful to compare to chronic muscle diseases in humans.

## Abbreviations

DMD: Duchenne muscular dystrophy; FLASH: Fast low angle shot MR imaging; FOV: Field of view; MIPAV: Medical image processing, analysis and visualization; MRI: Magnetic resonance imaging; RARE: Rapid acquisition with relaxation enhancement; WT: Wild-type; TR/TE: Repetition time/echo time.

## Competing interests

The authors declare that they have no competing interests.

## Authors’ contributions

SJPP and RML conception and design of research; SJPP, SX, and RML performed experiments; SJPP, SX, RJM, and RML analyzed data; SJPP, SX, RJM, and RML interpreted results of experiments; SJPP and RML prepared figures; RML drafted manuscript; SJPP, SX, RJM, and RML edited and revised manuscript; SJPP, SX, RJM, and RML approved final version of manuscript. All authors read and approved the final manuscript.
